# Using a new interrater reliability method to test the modified Oulu Patient Classification instrument in home health care

**DOI:** 10.1002/nop2.126

**Published:** 2018-02-04

**Authors:** Jill Flo, Bjørg Landmark, Ove Edward Hatlevik, Lisbeth Fagerström

**Affiliations:** ^1^ Faculty of Health and Social Sciences Department of Nursing and Health Sciences University College of Southeast Norway Drammen Norway; ^2^ Institute for Research and Development for Nursing and Care Services Drammen Norway; ^3^ Faculty of Education and International Studies Department of Primary and Secondary Teacher Education Oslo and Akershus University College of Applied Sciences Oslo Norway

**Keywords:** home health care, interrater reliability, nursing intensity, Oulu Patient Classification instrument, patient classification system

## Abstract

**Aim:**

To test the interrater reliability of the modified Oulu Patient Classification instrument, using a multiple parallel classification method based on oral case presentations in home health care in Norway.

**Design:**

Reliability study.

**Methods:**

Data were collected at two municipal home healthcare units during 2013–2014. The reliability of the modified OPCq instrument was tested using a new multiple parallel classification method. The data material consisted of 2 010 parallel classifications, analysed using consensus in per cent and Cohen's kappa. Cronbach's alpha was used to measure internal consistency.

**Results:**

For parallel classifications, consensus varied between 64.78–77.61%. Interrater reliability varied between 0.49–0.69 (Cohen's kappa), the internal consistency between 0.81–0.94 (Cronbach's alpha). Analysis of the raw scores showed 27.2% classifications had the same points, 39.1% differed one point, 17.9% differed two points and 16.5% differed ≥3 points.

## 1 INTRODUCTION

A gradual increase in life expectancy has resulted in a larger ageing population in developed countries and concern is growing about a probable healthcare professional deficit due to considerable demands for nursing resources in home health care (HHC) (European Union, Eurostat, 2016a, 2016b). An increased range of healthcare services will therefore be needed soon to meet the requirements of increasingly older populations. The number of available hospital beds is decreasing, with an evident shift towards beds in nursing homes, residential care facilities or HHC (European Union, Eurostat, 2016a, 2016b). To ensure good quality care, nurse managers need a workforce planning tool to follow‐up and monitor nursing intensity (NI) and the allocation of nursing resources. NI relates to how demanding a nursing situation is and how much care, help and support a patient has received (Fagerström, 1999; Morris, MacNeela, Scott, Treacy, & Hyde, 2007).

In hospital settings, a clear association between nursing resources (competence and numbers) and patient outcomes (patient safety and mortality) has been seen (Aiken, Clarke, Sochalski, & Silber, 2002; Aiken et al., 2014; Junttila, Koivu, Fagerström, Haatainen, & Nykänen, 2016). In nursing homes, fewer nursing hours have been associated with deficiencies (Harrington, Zimmerman, Karon, Robinson, & Beutel, 2000), while higher nursing hours show lower rates of pressure ulcers (Lee, Blegen, & Harrington, 2014). Corresponding studies in an HHC setting have not been found, but our supposition is that the correct allocation of nursing resources is crucial to ensuring quality care in such a setting.

Older and ageing populations have complex care needs (European Commission 2013, European Union, Eurostat, 2016a, 2016b) and there are many challenges involved in the realization of HHC. HHC services are fragmented and task‐oriented (Landmark, Aasgaard, & Fagerström, 2013), with patients experiencing delayed access to services, equipment supplies or medication and nursing staff experiencing unacceptable working conditions (Gautun & Bratt, 2014; Lang et al., 2014). Differences in staff competence and/or roles can also constitute a challenge in the allocation of nursing resources (Bing‐Jonsson, Hofoss, Kirkevold, & Bjørk, 2016; De Vliegher, Declercq, Aargeerts, & Moons, 2016; Flöjt, Hir, & Rosengren, 2014; Johansen & Fagerström, 2010; Luz & Hanson, 2015).

Measuring NI and the allocation of nursing resources is complex and several tools and patient classification systems (PCS) have been developed for use with older patients in HHC settings: e.g. the Clinical Care Classification (CCC) system (Saba, 2002), Resident Assessment Instrument (interRAI), Resource Utilization Groups (RUG III) (Carpenter & Hirdes, 2013), RAI‐HC (Toye, 2016), Community Health Intensity Rating scale (CHIRS), Easely‐Storefjell Patient Classification Instrument (R‐ESPCI) (Brady et al., 2007), Community Client Need Classification System (CCNCS) (Byrne, Brady, Horan, Macgregor, & Begley, 2007) and Caseload Intensity Tool (CIT) (Collister, Slauenwhite, Fraser, Swanson, & Fong, 2014). The Katz Index of Independence in Activities of Daily Living (Katz, Ford, Moskowitz, Jackson, & Marjorie, 1963) and the modified Katz ADL (Laan et al., 2014) measure functional ability and are well known. Some municipalities in Sweden use the Time in Care instrument (TiC) (Thorsell, 2011). In Norway, individual patients’ resources and needs for assistance are registered in a central health register (IPLOS), from which national statistics for nursing and care services are derived (Norwegian Directorate of Health 2013). Most of the above‐mentioned instruments primarily measure patients’ functional ability, not their psychological, social or spiritual needs nor the nursing care related to these. There is limited knowledge of NI in HHC and reliable instruments for measuring NI and nursing resources in such a setting are missing.

In the Nordic countries, the RAFAELA system is the most commonly used PCS. Used to measure NI and nurse staffing in hospital settings, the RAFAELA system is based on a holistic and person‐centred perspective, where balance is sought between each patient's individual care needs and the nursing resources needed to thereby guarantee good care for patients and good working conditions for staff (Andersen, Lønning, & Fagerström, 2014; Fagerström, 1999; Frilund, 2013; Pusa, 2007; Rauhala, 2008). Nurse managers can use the RAFAELA system to assure nursing quality, good patient outcomes and good working conditions for staff and to reduce sick leave among nurses (Junttila et al., 2016; Rauhala et al., 2007). It is an effective tool whereby resource allocation can be managed (Fagerström, Lønning, & Andersen, 2014; Fagerström & Rauhala, 2007). The RAFAELA system can be integrated into an organization's pre‐existing management or patient administrative system and has a positive effect on nurses’ clinical practice, which consequently influences patient outcomes (Fagerström et al., 2014).

The RAFAELA system is one of the few PCSs that meet the criteria for validity and reliability testing (Fasoli & Haddock, 2010). In the RAFAELA system, patients’ care needs are classified daily through the Oulu Patient Classification instrument (OPCq). The actual study was a part of a research project investigating the use of the RAFAELA system in a Norwegian HHC setting. The aim of this study was to test the reliability of the modified OPCq instrument in HHC using a new method, a multiple parallel classification method based on oral reports of patient cases.

### 1.1 Description of the OPCq instrument as part of the RAFAELA system

The RAFAELA system gives a professional overview of daily NI per patient and daily workload per nurse through the daily classification of patients’ care needs and daily registration of nursing resources. The RAFAELA system consists of the following components: 1. Daily registration of patients’ NI using the OPCq instrument; 2. Daily registration of actual nurse staffing resources; and 3. Determination of each unit's optimal NI level using the Professional Assessment of Optimal Care Intensity Level instrument (PAONCIL) (Rauhala & Fagerström, 2004; Rainio & Ohinmaa, 2005; Rauhala & Fagerström, 2007; Rauhala et al., 2007; Fagerström & Rainio, 1999; Fagerström et al., 2014; for a detailed description of the RAFAELA system, please see earlier research).

The OPCq instrument consists of six sub‐areas: 1. Planning and coordination of nursing care; 2. Breathing, blood circulation and symptoms of disease; 3. Nutrition and medication; 4. Personal hygiene and secretion; 5. Activity, sleep and rest; and 6. Teaching, guidance in care and follow‐up care, emotional support. In a hospital setting, nurses measure these sub‐areas at regular intervals once per calendar day, in an HHC setting after visiting the patient. Each sub‐area is scored from 1 to 4, with A = 1 point (a patient who manages more or less on his/her own), B = 2 points (a patient who occasionally is in need of care), C = 3 points (repeated need for care, complex) or D = 4 points (in need of continuous or very complex care and cannot manage unaided at all) (Fagerström, 1999; Fagerström, Rainio, Rauhala & Nojonen, 2000). The sum of these yields a raw score, which can vary from 6 to 24 and is the total NI points per patient per day. Higher scores indicate increased care and complexity levels. Patients are classified into five categories based on this raw score. Category 1: 6–8 points (minimal need for care), category II: 9–12 points (average need for care), category III: 13–15 points (more than average need for care), category IV: 16–19 points (maximum need for care) and category V: 20–24 points (intensive care required) (Fagerström, 2009; Rauhala & Fagerström, 2004). The resulting NI points can be recorded directly as raw scores or categories (I–V) (Rauhala & Fagerström, 2004; for a detailed description of the OPCq instrument, please see earlier research). The OPCq instrument used in the actual study was a modified version designed for use in an HHC setting (Flo, Landmark, Hatlevik, Tønnessen, & Fagerström, 2016). The modification of the OPCq instrument occurred as follows: the requirement that nursing staff assess electrolyte and acid–base disturbance or increased intracranial pressure was removed (sub‐area 1), patient positioning was changed to bedridden (sub‐area 2), management of prophylactic medication was changed to continuous medication (sub‐area 3) and the need for advice prior to discharge from hospital was removed (sub‐area 6). The key term “occasional” was adjusted to “need for occasional help” (sub‐areas 2–6).

A 2‐day introduction (educational programme) for registered nurses (RNs) and practical nurses (PNs) was held in October 2012 at the two HHC units included in the study by the Finish Consulting Group (FCG Ltd.) (2017). All subsequent and further education in relation to the project was the responsibility of the project leader. The assistants and students participating in the project were introduced to and trained in the use of the OPCq classification system in clinical practice by RNs or PNs.

According to RAFAELA system guidelines, the reliability of the OPCq should be tested annually at each unit where the system is in daily use using an independent parallel classifications by two nurses. The reliability of the OPCq instrument has been tested from various angles. Determined through consensus in per cent, the reliability of the instrument in hospital settings (category I–V) was on average 77% (Fagerström & Rauhala, 2007), with the main reliability value being 73.2% for 2006 and 78.7% for 2007 (Fagerström, 2009). In a study by Andersen et al. (2014), the reliability of the instrument using consensus in per cent varied between 70.1 and 89% and, using Cohen's kappa (k), variation in the patient categories was 0.59–0.81 and in the sub‐areas 0.45–0.90. In another study in a primary healthcare setting, a consensus in per cent of the parallel classifications varied between 66% and 77% (in total 71%), with Cohen's weighted kappa (Kw) 0.24–0.71 and Crohnbach's alpha 0.45–0.88 (Frilund & Fagerström, 2009). In a recent study in a hospital setting by Liljamo, Kinnunen, Ohtonen, and Saranto (2017), the results indicate that the consensuses in per cent for NI categories I–V was 70.8%, although a variation between periods was seen (50.5–93.2%). The Kw was 0.87 (varying between 0.40–0.96) and K 0.57 (varying between 0.27 and 0.87).

In all above‐mentioned studies, traditional parallel classifications have been used for reliability testing, that is two nurses caring for the same patient on the same day independently classify the patient's care needs and NI. Analyses of such classifications, used as the base for comparisons between two raters/nurses, have always previously been based on categories and not raw points, except the recent study by Liljamo et al. (2017).

## 2 METHODS

### 2.1 Aim

The aim of the study was to test the interrater reliability of the modified OPCq instrument, using a new multiple parallel classification method based on oral case presentations in home health care in Norway.

### 2.2 Design

The research design was based on interrater reliability testing, which is the extent of agreement among data collectors (McHugh, 2012). For the purposes of the actual study, a new multiple parallel classification method was developed. The guidelines for Reporting Reliability and Agreement Studies (GRRAS) (Kottner et al., 2011) were followed during reporting of the actual study.

### 2.3 Setting

Part of a municipal research and development programme, the study was realized in collaboration with a regional University College during 2012–2014. The study was conducted in two HHC units (A and B) in a medium‐size city, population about 70,000, in southeast Norway during 2013 and 2014. During the period of data collection, about 214 patients received nursing care through the two HHC units. In HHC in Norway, RNs, PNs and assistants provide nursing care and assist patients with personal activities of daily living (PADL). RNs are, however, more often responsible for acute care needs and specialized nursing interventions (Johansen & Fagerström, 2010). While RNs, PNs and assistants can help patients with daily household tasks, it is home aid workers who primarily bear the responsibility for such in patients’ homes. Due to the limited scope of their duties, home aid workers were not included in this study.

### 2.4 Participants

The participants consisted of RNs, PNs, assistants and students. Inclusion criteria were working at least 50% and during the day. Staff working night shifts were not invited to participate in the study. In HHC in Norway, RNs hold a bachelor's degree and are responsible for the planning and management of patients’ care and the supervision of other healthcare workers. PNs hold a vocational degree, provide basic nursing care and are typically supervised by RNs. Assistants, who are not required to hold any postsecondary degree and students at different levels also participated. A total of 67 participants conducted the parallel classifications and of these 19 (28.4%) were RNs, 26 (38.8%) PNs, 10 (14.9%) assistants and 12 (17.9%) students. Most of the participants had independently classified patients’ NI from between a couple of months to 1 year before participation in the study.

### 2.5 Data collection and development of a new method for parallel classification

A new *multiple parallel classification method* based on oral case presentations was developed, because the most common method for testing interrater reliability, parallel classifications with two independent raters (a main and a secondary rater) (McHugh, 2012) as used in hospital settings (Andersen et al., 2014; Fagerström, 2009; Fagerström & Rauhala, 2007), was deemed not feasible for use in an HHC setting. In HHC, nursing staff primarily work alone and it is therefore neither possible nor practical to use a method requiring two raters at the same time. Two nursing staff visiting the same patient during the testing period was deemed too costly/resource demanding, so a new method based on oral case presentations was developed.

The study periods were 4 November 2013–28 April 2014 at unit A and 9 December 2013–20 January 2014 and 6 February 2014–14 February 2014 at unit B, weekdays only. Each morning the nurse managers at the two units (A and B) selected one or two patient cases to be parallel classified during the shift and also determined the main rater. The nurse managers were responsible for an even distribution of patient cases concerning background variables (age, gender), care needs and NI. After visiting the selected patient, the main rater (RN, PN, assistant or student) classified NI using the modified OPCq instrument. For practical reasons, the parallel classifications were performed by the secondary raters the same day during their lunch break. The secondary raters did not visit the actual patient, so the classifications were based on the main rater's oral case presentation. A special structure was developed for the oral case presentations.

The main rater presented the patient case in accordance with a delineated structure, including the variables age, gender, diagnoses, problems or needs, observations, performed nursing activities, and treatments during the HHC visit. The main raters’ NI classifications and scores were kept from the secondary raters. After the main rater's presentation, 3–10 secondary raters were asked to independently classify the patient's NI without communicating, discussing or exchanging information with one another during the process; only clarifying questions were allowed. During the study periods, participants could act as main or secondary raters several times. A classification form was used for all classifications, with the main rater collecting all forms after each parallel classification and giving them to the nurse managers at the HHC unit. The respective nurse managers then collected all forms and distributed them to the project leader.

### 2.6 Ethical considerations

The Norwegian Social Science Data Services (NSD) provided approval prior to commencement of the study. Appropriate permission from the municipality was sought and given for the study, likewise a license from the Finish Consulting Group (FCG) giving the municipality permission to use the RAFAELA system. The nurses in the study gave informed consent. The patients received nursing care through the two HHC units as previously planned during the project period and because all patient data were anonymized, no informed consent was required from them.

### 2.7 Statistical analyses

The interrater reliability method (McHugh, 2012) was used to analyse the data: consensus in per cent and Cohen's kappa and Cronbach's alpha were used to measure internal consistency (Pallant, 2015; Polit & Beck, 2014). The calculation of consensus as per cent agreement and Cohen's kappa were in raw scores instead of categories I–V. Raw scores are more sensitive than categories and therefore more correct and reliable. For reliability analyses, the steering group of the RAFAELA system in Finland has indicated a preference for the use of raw scores.

The interrater reliability method was used to test interrater agreement of the OPCq sub‐areas. Consensus in per cent was used for the parallel classifications as this is easy to calculate, is directly interpretable and allows the identification of possibly problematic variables (McHugh, 2012). In a hospital setting, the recommendation is ≥70% consensus (Fagerström & Rauhala, 2007; Rauhala et al., 2007). However, consensus in per cent does not make allowances for the possibility that raters may guess when rating some variables due to uncertainty (McHugh, 2012). Cohen's kappa was calculated for every main rater compared with every secondary rater, i.e. each RN, PN, assistant or student rating the same patient case. Of the 53 patient cases rated, differences between 3 and 10 secondary raters were seen.

While Cohen's kappa does take into account the possibility of guessing among multiple data collectors, it is by far the most used measure of agreement (McHugh, 2012; Veierød, Lydersen, & Laake, 2012). Cohen's kappa is an important supplement to consensus in per cent and is a robust statistical method. Kappa can range from −1 to +1, where 0 represents agreement that can be expected from random chance and +1 represents perfect agreement (Altman, 1999). As recommended (Altman, 1999 guidelines; Anthony, 1999; Kirkwood & Sterne, 2003), Landis and Koch's (1977) guidelines were followed. The kappa results were interpreted as follows: values ≤0 no agreement, 0.01–0.20 none to slight, 0.21–0.40 fair, 0.41–0.60 moderate, 0.61–0.80 substantial and 0.81–1.00 almost perfect (Landis & Koch, 1977). As noted by McHugh (2012), while 80% agreement is recommended, because kappa values 0.41–0.60 are considered moderate, the lowest value 0.40 (k) may be considered adequate. Still, McHugh (2012) suggested that any kappa lower than 0.60 indicates inadequate agreement. According to De Vet, Mokkink, Terwee, Hoekstra, and Knol (2013), kappa is a relative measure and not sufficiently informative; it is a measure of reliability, not agreement and not recommended for use in measuring observer variation in clinical practice. A low kappa value may not always be indicative of low agreement according to Gisev, Bell, and Chen (2013). Nevertheless, in this study both consensus in per cent and Cohen's kappa were used to make the results more comparable with previous studies (Andersen et al., 2014; Fagerström, 2009; Fagerström & Rauhala, 2007; Frilund & Fagerström, 2009).

Cronbach's alpha is widely used to measure the internal consistency of an instrument (Polit & Beck, 2014), and in this study, it was used to estimate the reliability of the modified OPCq instrument when testing in a new context, HHC. In relation to scales, internal consistency refers to whether items ‘hang together’ (Pallant, 2015) and the less variation seen in repeated measurements, the higher an instrument's reliability. A commonly accepted rule for describing internal consistency when using Cronbach's alpha is: α ≥ 0.9 = excellent, 0.9 > α ≥ 0.8 = good, 0.8 > α ≥ 0.7 = acceptable, 0.7 > α ≥ 0.6 = questionable, 0.6 > α ≥ 0.5 = poor, 0.5 > α = unacceptable (George & Mallery, 2003). While values above 0.7 are acceptable, values above 0.8 are preferable (Pallant, 2015).

In this study, a research assistant entered the parallel classifications (the scores) into an Excel (Microsoft office) database. The data were then transferred into an IBM Package for Social Sciences (SPSS) Statistics Version 23 database.

## 3 RESULTS

A total of 2010 parallel classifications (335 * 6 sub‐areas) took place during the period November 2013–February 2014. A total of 53 patient cases were classified by the main raters into the following categories: category I: 6 (11.3%); category II: 24 (45.3%); category III: 11 (20.8%); category IV: 11 (20.8%) and category V: 1 (1.9%). The majority of patient cases were classified into classes II, III and IV, indicating average, more than average or maximum need for care.

Of the 53 patient cases/patients, the background variable data for 44 patients (83%) were available. The remaining nine had either moved to nursing homes/residential homes or passed away. Most patients (*N* = 44) were female, 30 (68.2%) and 14 (31.8%) were male. The mean age was 83 years (median* *= 84 years, *SD* 9.6), with patients aged 48–101 years. A complex patient health status was seen, and several had chronic diagnoses.

Of the 335 classifications, 91 (27.2%) had the same raw scores. Disagreement was one point in 131 classifications (39.1%), two points in 60 (17.9%) and three points or over in 53 (15.9%) (Table 1).

**Table 1:** Classification based on raw scores and differences in points

Raw score/points	*N*	%
0 point difference in raw score	91	27.2
1 point difference in raw score	131	39.1
2 point difference in raw score	60	17.9
3 point difference in raw score	31	9.3
4 point difference in raw score	12	3.6
5 point difference in raw score	6	1.8
6 point difference in raw score	2	0.6
7 point difference in raw score	2	0.6
Total	335	100.0

The consensus in per cent of the parallel classifications for sub‐areas 1–6 was 64.78%–77.61%. (Table 2). Cohen's kappa showed an interrater reliability of 0.49–0.69 (Table 2). The highest consensus was found for sub‐area 4 (Personal hygiene and secretion) (77.61%, k 0.69). Sub‐area 6 (Teaching, guidance in care and follow‐up care, emotional support) showed a weaker consensus (64.78%) and the lowest kappa (k 0.49). For sub‐areas 1–6, Cronbach's alpha was 0.81–0.94 (Table 2). Good internal consistency was seen for sub‐areas 1, 2, 3, 5 and 6, while sub‐area 4 had excellent internal consistency (Table 2).

**Table 2:** Parallel classifications, sub‐areas 1‐6 of the OPCq instrument, consensus in per cent, Cohen's kappa and Cronbach's alpha

Sub‐areas	Consensus %	Cohen's kappa	Cronbach's alpha
1. Planning and coordination of nursing care	70.45%	0.56	0.84
2. Breathing, blood circulation and symptoms of disease	70.45%	0.52	0.81
3. Nutrition and medication	73.43%	0.61	0.87
4. Personal hygiene and secretion	77.61%	0.69	0.94
5. Activity, sleep and rest	71.64%	0.57	0.83
6. Teaching, guidance in care and follow‐up care, emotional support	64.78%	0.49	0.85

Using a calculation of the total raw scores for sub‐areas 1–6 of the OPCq instrument, the consensus was 71%. Using a calculation for patient categories I–V, the kappa was 0.60, which according to McHugh (2012) indicates adequate agreement. Here, in that a difference of 1 point in total is considered a deviation, the kappa is deemed acceptable even though lower than usual.

## 4 DISCUSSION

Using a new multiple parallel classification method, we tested the interrater reliability of the modified OPCq instrument in two HHC units in a Norwegian municipality. We found slightly lower consensus in per cent than in a study conducted in Finland in primary health care (≥70%) (Frilund & Fagerström, 2009) or in other studies in hospital settings (≥70%) (Andersen et al., 2014; Fagerström, 2009; Fagerström & Rauhala, 2007; Liljamo et al., 2017).

The calculations here were based on raw scores, a method which is more sensitive and perhaps more accurate than in previous studies, which have calculations based on categories (I–V). In our results, we see that 282 (84.2%) classifications differed from zero to two points, while only 53 (15.9%) differed over three points, this is slightly higher results than the study of Liljamo et al. (2017). When calculations are based on categories (I–V), classifications can differ up to four points while agreement and interrater reliability remain constant. In earlier studies (Andersen et al., 2014; Fagerström, 2009; Fagerström & Rauhala, 2007; Frilund & Fagerström, 2009), patient categories, not raw NI points, were used in the calculation of both percentage agreements and interrater reliability. Thus, this should be taken into consideration when comparing the results of the actual study with earlier studies.

Here the agreement shows a consensus in per cent of 64.78%–77.61% and Cohen's kappa indicating moderate to slight agreement according to Landis and Koch (1977). Cronbach's alpha was interpreted as good and excellent (Table 2). While these are slightly lower results than those seen in a study by Frilund and Fagerström (2009), that study had a healthcare centre setting and not an HHC setting and moreover only included RNs and PNs.

In this study, disagreement was greatest in relation to the classifications of sub‐area 1 (Planning and coordination of care), sub‐area 2 (Breathing, blood circulation and symptom of disease), sub‐area 5 (Activity, sleep and rest) and sub‐area 6 (Teaching, guidance/follow‐up care and emotional support). We concluded that these sub‐areas are more difficult for nurses to assess than sub‐areas 3 (Nutrition and medication) and 4 (Personal hygiene and secretion), which is consistent with earlier findings (Andersen et al., 2014; Fagerström et al., 2000; Frilund & Fagerström, 2009; Liljamo et al., 2017). Sub‐area 4 had the highest consensus and a substantial agreement according to McHugh (2012); this is acceptable. We interpret the Cronbach's alpha of sub‐area 4 as being excellent and indicative of care needs well known to nurses. This is also in line with similar findings in earlier studies (Andersen et al., 2014; Fagerström et al., 2000; Frilund & Fagerström, 2009).

The lowest agreement was seen in sub‐area 6. The difficulties that nurses have when assessing this sub‐area can emanate from different sources, such as decisions that a municipality has made in regard to care plans; sub‐area 6 might not be prioritized in a delineated care plan. Also, according to Tønnessen, Nortvedt, and Førde (2011), nurses ration care due to time constraints, consequently prioritizing medical or physiological needs over psychosocial and spiritual needs. McCormack and McCain (2010) maintain that providing holistic care is essential in a person‐centred process, yet time constraints can hinder such. Sub‐areas 1 and 5 showed a consensus slightly above the recommended level (>70%) and a kappa of 0.56–0.57. According to Landis and Koch (1977), this kappa indicates moderate agreement, while McHugh (2012) argues that kappa below 0.60 indicates inadequate agreement. Sub‐areas 1 and 5 can be difficult for nurses in HHC to assess because each patient visit is short, making an overview of the situation problematic. Another aspect is that RNs are tasked with the planning and coordination of HHC care but PNs, assistants and students are not. In sub‐area 2, consensus was slightly above 70% but kappa showed a moderate agreement according to Landis and Koch (1977). Of the study participants, only 28.4% were RNs, while the remainder were PNs, assistants or students, which likely influenced the classifications in this sub‐area.

This study was a part of a larger research project where participants assessed the educational programme overseen by the FCG and the project leader as being good (Flo et al., 2016). Different educational and staff competence levels in HHC (Bing‐Jonsson et al., 2016) probably influenced the participants’ understanding of the different classification levels. In future, the possibility to regularly discuss the sub‐areas, different levels A‐D and keywords together with colleagues is recommended. Training in classifying and regular practice in performing parallel classifications may positively influence common understanding of the different classification levels.

One probable limitation of the multiple parallel classification method used in this study is that, on the day of classification, only the main rater met the patient being classified. If when using the OPCq instrument the main rater did not properly follow the delineated structure for describing nursing care, variation will be seen between the main and secondary raters’ classifications. We surmise therefore that it would be more reliable if both main and secondary raters actually met the patient on the day of classification, but this is not possible in an HHC setting. For parallel classifications, it would even be possible to gather the secondary data from patient records (Altafin et al., 2014; Liljamo et al., 2017; Stafseth, Tønnessen, Diep, & Fagerström, 2017). Nevertheless, that method also has its limitations in that nursing documentation, especially in Norwegian HHC, can be considered inconsistent and of variable quality.

In a study by Kottner, Halfens, and Dassen (2010) on the use of the care dependency scale (CDS) in HHC, the nurse primarily responsible for the selected patient's care completed the first classification while a different nurse performed the second classification 1–3 days later. Given that we assume that care needs fluctuate continuously, we developed a new method of interrater reliability testing to ensure that classifications occurred on the same day. It will also probably be valuable in future studies to ensure that the main rater is an RN or PN and has adequate experience of working in an HHC setting.

The population of older and fragile people is growing, as is their need for care. Hasseler, Görres, Altmann, and Stolle (2006) maintained that a gap exists between the provision of nursing services and the need for care. In the care environment in a person‐centred approach, a focus should exist on the context where care is delivered and the factors that should be taken into consideration should include, among other things: appropriate skill mix, supportive organizational systems and effective staff relationships (McCormack & McCance, 2010). To meet the requirements for implementing person‐centred care, managers need access to systems that help them with the allocation of staff resources. The RAFAELA system, of which the OPCq instrument is part, enables the allocation of nursing resources in accordance with patients’ care needs and safety during a certain period of time (Fagerström et al., 2014).

## 5 LIMITATIONS

There is limited information about the participant background variables, such as working experience, etc. Nurses with different educational backgrounds may interpret patients’ NI differently, especially those without postsecondary degrees. In other studies on interrater reliability, the various individuals collecting data may experience and interpret the data differently (McHugh, 2012). In this study, all participants participated in a training programme and learnt how to use the OPCq instrument prior to participation. They furthermore, according to guidelines (Kottner et al., 2011), had performed classifications using the OPCq instrument by themselves to ensure that they were sufficiently trained prior to participation. In future studies, participants’ clinical backgrounds and work experience should be investigated, because these factors may heavily influence reliability and agreement estimates (Kottner et al., 2011). In this study, the patient cases included mainly older patients with different care needs. It is important to specify the data on the subject population of interest, according to Kottner et al. (2011) and as such this could have been more well specified in this study, including e.g. diagnosis, stages of disease, assistance, aid requirements and/or length of time receiving HHC services.

## 6 CONCLUSION

The investigation of this new, multiple parallel classification method that is based on oral case presentation shows that this is a method that can be used in HHC when parallel classification with two independent raters is not feasible.

The results seen here are slightly lower than those seen in previous studies conducted in primary healthcare and hospital settings. A total raw score was used in the calculations in this study, versus other studies where patient categories I‐V are used, except one recent study in hospital setting used raw score, which makes comparisons somewhat difficult. While participants’ assessments of the different sub‐areas were in line with previous studies, some sub‐areas may need improvement to better correspond to an HHC setting. For those that showed low agreement here, more detailed description in the RAFAELA manual is needed. As this study was based on a small sample, a need exists for additional research.

## CONFLICT OF INTEREST

No conflict of interest has been declared by the authors.

## AUTHOR CONTRIBUTIONS

All authors meet at least one of the following criteria, as per ICMJE recommendations (http://www.icmje.org/recommendations/), and have agreed on the final version.

- substantial contribution to the conception and design of the article, acquisition of data or analysis and interpretation of data;
- drafting or critical revision of the article for important intellectual content.
